# Dual Roles of HbA1c Variability and Body Composition for Cardiovascular Risk: A Cohort Study of 8224 Adults With Type 2 Diabetes Mellitus

**DOI:** 10.1002/jcsm.70028

**Published:** 2025-07-29

**Authors:** Sunyoung Kim, Jiyoung Hwang, Dong Keon Yon, Hyunji Sang, Selin Woo, Ho Geol Woo, Hyunjung Lim, Jungha Park, Fei‐Yuan Hsiao, Liang‐Kung Chen, Sang Youl Rhee

**Affiliations:** ^1^ Department of Family Medicine, Kyung Hee University Medical Center Kyung Hee University College of Medicine Seoul South Korea; ^2^ Center for Digital Health, Medical Science Research Institute Kyung Hee University College of Medicine Seoul South Korea; ^3^ Department of Pediatrics, Kyung Hee University Medical Center Kyung Hee University College of Medicine Seoul South Korea; ^4^ Department of Endocrinology, Kyung Hee University Medical Center Kyung Hee University College of Medicine Seoul South Korea; ^5^ Department of Neurology Kyung Hee University College of Medicine Seoul South Korea; ^6^ Department of Medical Nutrition, Graduate School of East‐West Medical Science Kyung Hee University Yongin South Korea; ^7^ Graduate Institute of Clinical Pharmacy, College of Medicine National Taiwan University Taipei Taiwan; ^8^ School of Pharmacy, College of Medicine National Taiwan University Taipei Taiwan; ^9^ Department of Pharmacy National Taiwan University Hospital Taipei Taiwan; ^10^ Center for Healthy Longevity and Aging Sciences National Yang‐Ming Chiao Tung University Taipei Taiwan; ^11^ Center for Geriatrics and Gerontology Taipei Veterans General Hospital Taipei Taiwan; ^12^ Taipei Municipal Gan‐Dau Hospital Managed by Taipei Veterans General Hospital Taipei Taiwan

**Keywords:** body composition, cardiovascular disease, diabetes mellitus, HbA1c variability, skeletal muscle mass

## Abstract

**Background:**

Glycaemic variability and body composition are emerging predictors of cardiovascular disease (CVD) in patients with Type 2 diabetes mellitus (T2DM); however, their combined impact remains unclear. We investigated the association among HbA1c variability, body composition parameters and cardiovascular outcomes in adults with T2DM.

**Methods:**

This retrospective cohort study analysed electronic health records from a university hospital (2011–2020), including 8224 adults (mean age 58.3 years, 50.1% women) with T2DM and no history of CVD. HbA1c variability score (HVS) was defined as the percentage of successive measurements differing by ≥ 0.5% (5.5 mmol/mol). Body composition was assessed by bioimpedance analysis. The primary outcome was incident CVD (ischemic heart disease, heart failure, atrial fibrillation, stroke, myocardial infarction).

**Results:**

During median follow‐up of 4.0 years, patients with high HVS (third tertile) showed significantly increased CVD risk compared to low HVS (first tertile) (adjusted hazard ratio [aHR] 1.70 [95% CI 1.13–2.40]; *p* < 0.010). HbA1c variability demonstrated superior cardiovascular risk prediction over fasting and postprandial glucose variability. Individuals with high HVS had significantly higher systolic blood pressure (122.72 ± 14.96 vs. 120.53 ± 14.52 mmHg, *p* = 0.017), HbA1c (7.72% ± 1.75% vs. 7.02% ± 1.09%, *p* < 0.001) and lower skeletal muscle mass (24.60 ± 5.73 vs. 25.85 ± 7.84 kg, *p* < 0.001). Higher appendicular skeletal muscle mass was protective against CVD (aHR 0.75 [95% CI 0.63–0.88]), while increased total fat percentage elevated CVD risk (aHR 1.10 [95% CI 1.03–1.20]). HVS correlated positively with changes in total fat percentage (*β* = 0.439, *p* < 0.001) and negatively with changes in relative appendicular skeletal muscle mass (*β* = −0.258, *p* < 0.001). In multivariate analysis, significant contributors to increased CVD risk included high HVS (aHR 1.65, *p* = 0.011), elevated average HbA1c (aHR 1.09, *p* = 0.016) and age over 65 years (aHR 1.61, *p* = 0.035).

**Conclusions:**

HbA1c variability and body composition independently predicted cardiovascular outcomes in patients with T2DM. Higher HbA1c variability increased CVD risk by 70%, while higher appendicular muscle mass reduced risk by 25%, and higher total fat percentage increased risk by 10%. Incorporating these parameters into risk stratification models could enhance cardiovascular risk prediction and guide preventive strategies for diabetes management.

## 1 Introduction

Cardiovascular disease (CVD) remains the leading cause of mortality and morbidity in Type 2 diabetes mellitus (T2DM), posing a substantial global public health challenge and economic burden across nations of varying developmental status [1]. The complex interplay between diabetes and cardiovascular complications has prompted extensive research into risk prediction and prevention strategies. While the association between glycaemic control and diabetic complications is well established [2], traditional markers, such as glycated haemoglobin (HbA1c), may inadequately capture the dynamic nature of glycaemic fluctuations that influence vascular outcomes [3].

Recent evidence suggests that glucose variability, particularly HbA1c variability score (HVS), could independently predict adverse cardiovascular outcomes [3, 4, 5]. HVS might better reflect the pathophysiological mechanisms underlying vascular complications through unstable glycaemic patterns, hypoglycaemia‐induced oxidative stress and chronic inflammation [6, 7]. Elevated HVS has been associated with increased risks of CVD, possibly driven by unstable glycaemic patterns, hypoglycaemia‐induced oxidative stress, and inflammation [7]. Despite its potential clinical significance, current diabetes management guidelines primarily focus on average glycaemic control rather than glycaemic variability. The relationship between glycaemic fluctuations and cardiovascular risk remains incompletely understood, particularly in diverse populations with varying diabetes durations and treatment regimens [8, 9].

Beyond glycaemic parameters, emerging evidence highlights the crucial role of body composition in cardiometabolic risk stratification [10]. Traditional adiposity measures, such as body mass index (BMI), fail to differentiate between metabolically distinct tissues—fat mass (FM) and skeletal muscle mass (SMM) [11, 12, 13]. These components exhibit opposing effects on cardiovascular risk, with FM showing positive associations and SMM demonstrating potential protective effects. The interplay between visceral adiposity, skeletal muscle mass and glucose metabolism suggests a complex relationship that warrants further investigation. Moreover, recent studies have indicated that changes in body composition over time may have significant implications for cardiovascular outcomes independent of traditional risk factors.

Current risk prediction models for cardiovascular complications in T2DM primarily rely on conventional factors such as blood pressure, lipid profiles and smoking status. However, these models often have limited predictive accuracy, particularly in specific patient subgroups. Incorporating novel biomarkers and physiological parameters could potentially enhance risk stratification and guide personalized preventive strategies. Hence, we hypothesized that integrating novel metrics of glycaemic variability with detailed body composition analysis could provide more precise cardiovascular risk stratification in patients with T2DM.

This study aimed to evaluate the association between HbA1c variability and cardiovascular outcomes, investigate the modulatory role of body composition parameters, and assess their combined prognostic value in predicting cardiovascular risk in individuals with T2DM. Our findings have important implications for clinical practice by identifying novel risk factors and potential therapeutic targets for cardiovascular prevention in diabetes care.

## 2 Methods

### 2.1 Study Design and Participants

We conducted a retrospective longitudinal cohort study using electronic health records (EHRs) from Kyung Hee University Medical Center. The study period spanned from 1 January 2008 to 31 December 2022, allowing up to 14 years of follow‐up. Cohort entry events were defined as the baseline and follow‐up periods.

### 2.2 Cohort Entry Event

A cohort entry event was defined as a diagnosis of T2DM [14]. The study team developed a two‐stage subject selection process to reference the T2DM diagnosis. The two stages were (1) whether the patient had an International Classification of Diseases, 10th Revision (ICD‐10) code (E11.X) for T2DM and (2) whether there were any glucose‐lowering medications (GLMs) in the medication record other than metformin [15, 16]. Once participants were selected, their index date was the earliest recorded T2DM diagnosis or the first GLM prescription date.

### 2.3 Inclusion and Exclusion Criteria

**FIGURE 1:**
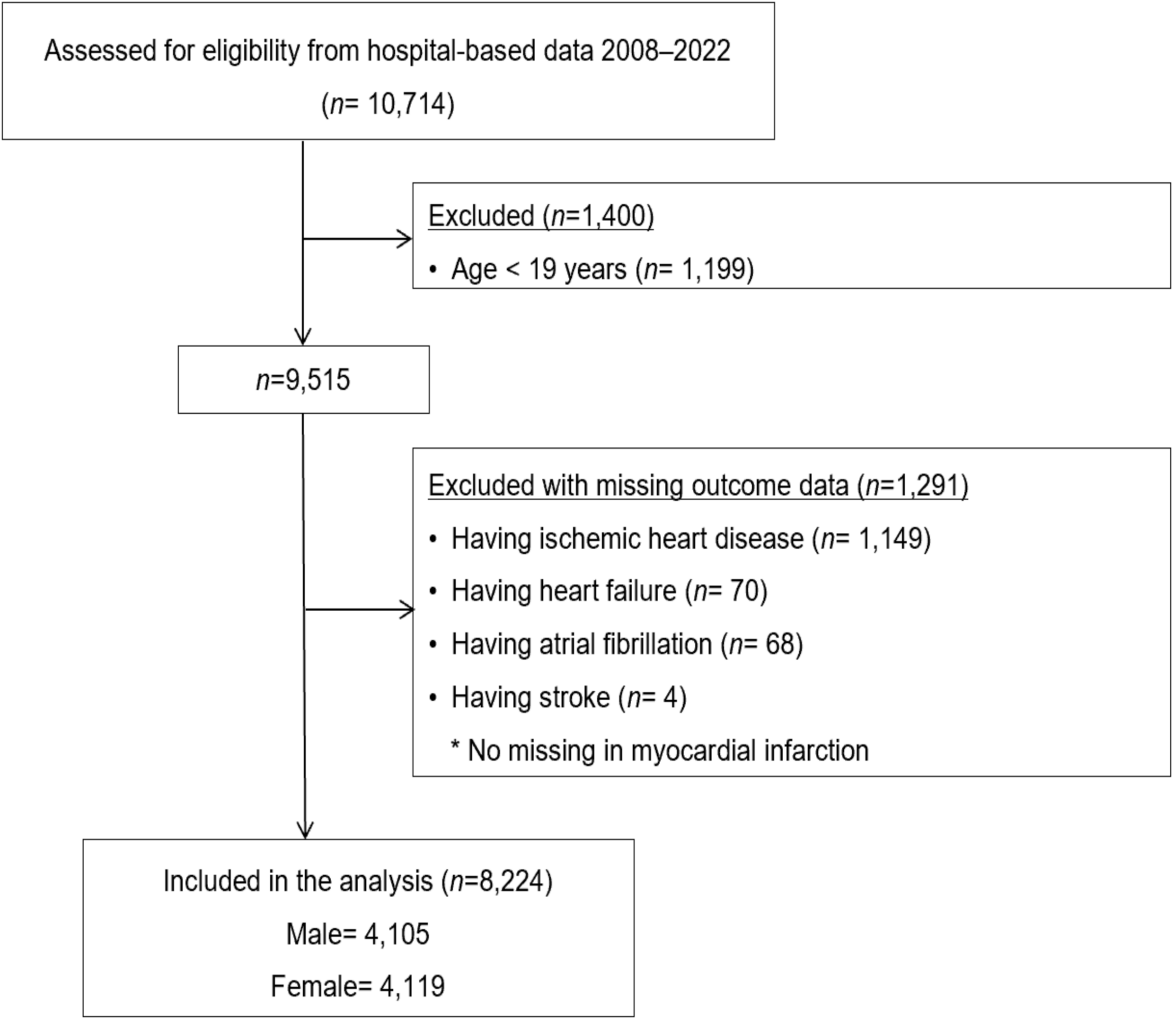
Schematic of the analysis pipeline.

Three inclusion criteria refined the initial event cohort: (1) age ≥ 19 years at baseline, (2) documented HbA1c measurements during the investigation window and (3) no prior medical history of ischemic heart disease, heart failure, atrial fibrillation and/or stroke diagnosis during the study period (Figure 1). Participants with a history of these preexisting cardiovascular conditions were excluded to prevent confounding and ensure that the analysis focused on new‐onset CVD in individuals with T2DM. Additionally, participants with missing data on primary outcomes (ischemic heart disease, heart failure, atrial fibrillation and stroke) were excluded from the analysis to ensure the robustness of the results.

### 2.4 Qualifying Cohorts

Eligible participants in the final study cohort required at least one HbA1c measurement within the first year of T2DM diagnosis and at least three HbA1c measurements during the follow‐up period. Furthermore, participants were required to have a patient encounter record > 365 days after post‐T2DM diagnosis. Those without encounter records > 365 days were considered incident cases, indicating insufficient longitudinal information for the study inclusion [17]. Individuals with only emergency department visits and/or inpatient stays ≥ 365 days from the index date and only outpatient visits to capture patients with regular follow‐ups were also excluded. Additionally, a loyalty cohort was established to minimize missing data and biases and to provide a meaningful cohort.

### 2.5 Cohort Exit Event

Participants exited the cohort based on one of the following: (1) observation of CVD–primary endpoint, (2) disenrollment–reasons for disenrollment could be relocation or transition to a different healthcare system outside the current EHR system or (3) right censoring–CVD not observed before the last recorded encounter or before the study cutoff date of 31 December 2022.

### 2.6 Demographic Data and Functional Assessment

Baseline demographic characteristics included age, sex and BMI. The baseline age was calculated by subtracting the birth date from the T2DM diagnosis date. Clinical and medication data were also reviewed, including comorbidities, complications (identified using ICD‐9/10 codes) and GLM use within 90 days of T2DM diagnosis.

### 2.7 Body Composition

Body composition was assessed by bioelectrical impedance analysis (BIA) (InBody720; Biospace Inc., Seoul, South Korea). Participants adhered to standardized conditions, such as fasting for 3 to 4 h, using the restroom, wearing light clothing and removing metal items, to ensure the reliability and accuracy of measurement outcomes. BIA measurements were recorded every 3–12 months during outpatient visits following the T2DM diagnosis, and these data were utilized for the analysis.

The measurements included the lean mass, fat mass, visceral fat area (VFA, cm^2^) and total fat percentage (TFP). Appendicular SMM (ASM) was calculated as the sum of lean tissue mass from all four limbs, and relative ASM (RASM) was calculated by dividing ASM by height squared (m^2^) [18]. Additional indices calculated to evaluate body composition included skeletal muscle index (SMI) = SMM/height^2^, FM index (FMI) = total FM/height^2^, visceral fat index (VFI) = VFA/height^2^, appendicular muscle‐to‐fat ratio (aMFR) = ASM/total body FM and total muscle‐to‐fat ratio (tMFR) = total body muscle mass/total body FM [18, 19].

### 2.8 Other Variables

Baseline systolic blood pressure (SBP) and diastolic blood pressure (DBP) were averaged from readings obtained during the first year post‐diagnosis to assess baseline SBP and DBP control. Laboratory data, including postprandial 2‐h glucose (PP2hrs), fasting plasma glucose (FPG), fasting blood glucose, HbA1c, total cholesterol, triglyceride, high‐density lipoprotein cholesterol, low‐density lipoprotein cholesterol, creatinine, aspartate transaminase, alanine transaminase, gamma‐glutamyl transferase and alkaline phosphatase levels, were collected. Renal function was assessed using the estimated glomerular filtration rate (eGFR), which was calculated using the CKD‐EPI (Chronic Kidney Disease Epidemiology Collaboration) equation. eGFR values were categorized into three groups: ≥ 60, 30–59 and < 30 mL/min/1.73 m^2^, representing normal/mild, moderate and severe renal impairment, respectively.

### 2.9 Outcome Definitions

New‐onset CVD among patients with T2DM was identified using ICD‐10 codes for five conditions: (1) ischemic heart disease (I20.X–I25.X), (2) heart failure (I50.X), (3) atrial fibrillation (I48.X), (4) stroke (I60.X–I69.X) and (5) myocardial infarction (I21.X–I22.X).

### 2.10 Statistical Analysis

Descriptive statistics were used to comprehensively present the cohort demographics. Continuous variables were expressed as mean ± standard deviation, while categorical variables were presented as percentages. Visit‐to‐visit variability in HbA1c was quantified using the HVS, calculated as the number of successive HbA1c values that differed by ≥ 0.5% (5.5 mmol/mol) from the previous measurement, divided by the total number of comparisons and multiplied by 100. For example, for four values (5.6%, 6.4%, 6.0% and 7.0%), two out of three comparisons would exceed the threshold, yielding an HVS of 66.7% [20]. Glycaemic variability in FPG and PP2hrs was assessed using the coefficient of variation (CV), calculated as the standard deviation divided by the mean of all available outpatient measurements collected during follow‐up. Participants were classified into tertile groups based on the actual distribution of CV values observed in the dataset for each variable. Pearson correlation coefficients were used to examine the relationships and changes between baseline and post‐treatment values for age, BMI, HbA1c, PP2hrs, FPG and body composition data obtained through BIA. In this study, baseline was defined as the earliest measurement obtained within 1 year of T2DM diagnosis. Post‐treatment referred to the follow‐up period after the initiation of glucose‐lowering therapy, during which subsequent measurements were used for variability analysis. Kaplan–Meier survival analysis and univariate and multivariate Cox regression analyses were conducted to evaluate the determinants of CVD incidence among patients with T2DM. Kaplan–Meier survival analysis was used to visualize the cumulative risk plots for each variable, and log‐rank tests were performed to compare survival differences between the groups. Univariate Cox regression analysis was used to assess the independent impact of each variable on CVD risk, whereas multivariate Cox regression analysis was adjusted for potential confounders to identify the determinants of CVD incidence. Additionally, multivariate Cox regression analysis was employed in the subgroup analyses of body composition to assess the relative effects of muscle and fat on CVD risk. A cubic spline analysis with three knots (25th, 50th and 75th percentiles) was performed to evaluate the linearity between the HVS and CVD risk. All statistical tests were two‐tailed, and the level of statistical significance was set at *p* < 0.05. Analyses were conducted using SAS software (Version 9.4) and R Version 4.4.2. In SAS, descriptive statistics were performed using PROC MEANS, Pearson correlation analysis was conducted with PROC CORR and Cox regression analysis was performed using PROC PHREG. In R (Version 4.4.2), Kaplan–Meier survival curves were generated using the survival package for Cox regression and survival analysis, and ggplot2 was used for visualization.

## 3 Results

### 3.1 Baseline Characteristics

As of 30 December 2022, the Kyung Hee University EHR database has identified 10 714 patients diagnosed with T2DM. After excluding individuals with preexisting conditions, 8224 participants were included in the final cohort (Figure 1). The cohort's mean age was 58.3 years, and women accounted for 50.1% of the population. Baseline assessments indicated an average blood glucose level of 131.53 mg/dL, HbA1c of 7.29% and an HVS of 0.42% ± 0.34%. The cut‐off values used to define the tertiles were 11.75 and 28.41 for HVS, 6.13 and 14.87 for PP2hrs variability and 3.85 and 9.93 for FPG variability. Upon stratifying HVS into tertiles, comparative analysis revealed that participants in the higher HVS group had significantly higher mean values for SBP (122.72 ± 14.96 vs. 120.53 ± 14.52 vs. 122.56 ± 76.04 mmHg; *p* = 0.017), HbA1c (7.72% ± 1.75% vs. 7.12% ± 1.33% vs. 7.02% ± 1.09%; *p* < 0.001), PP2hr glucose (158.20 ± 76.33 vs. 150.92 ± 68.24 vs. 146.10 ± 62.95 mg/dL; *p* < 0.001), fasting glucose (133.14 ± 51.00 vs. 131.88 ± 53.33 vs. 126.83 ± 45.99 mg/dL; *p* = 0.006), triglycerides (168.58 ± 84.86 vs. 168.38 ± 82.93 vs. 155.53 ± 75.81 mg/dL; *p* < 0.001) and total body fat percentage (31.74 ± 8.62 vs. 31.20 ± 8.19 vs. 30.82 ± 8.50; *p* = 0.000). In contrast, the mean values for creatinine (0.77 ± 0.35 vs. 0.83 ± 0.52 vs. 0.84 ± 0.66 mg/dL; *p* < 0.001) and SMM (24.60 ± 5.73 vs. 25.38 ± 5.78 vs. 25.85 ± 7.84; *p* < 0.001) were significantly lower in higher HVS groups (Table 1).

**TABLE 1:** Sociodemographic and lifestyle characteristics of the study population.

	HVS^a^			
	1st tertile (*n* = 2689)	2nd tertile (*n* = 2796)	3rd tertile (*n* = 2739)	*p*
Mean HVS	4.82 ± 2.90	20.75 ± 4.92	51.42 ± 8.26	< 0.001
Median HVS (range)	0.00 (0.00–11.75)	22.22 (16.70–28.41)	50.03 (37.08–100.00)	
Demographic characteristics				
Age, years	57.72 ± 12.42	60.30 ± 11.96	56.86 ± 11.80	< 0.001
Sex, females	1244 (46.26)	1505 (53.83)	1370 (50.02)	< 0.001
Laboratory data				
Systolic BP, mmHg	120.53 ± 14.52	122.56 ± 76.04	122.72 ± 14.96	0.017
Diastolic BP, mmHg	76.04 ± 9.85	75.49 ± 9.50	75.34 ± 9.58	0.355
HbA1c, %	7.02 ± 1.09	7.12 ± 1.33	7.72 ± 1.75	< 0.001
PP2hrs	146.10 ± 62.95	150.92 ± 68.24	158.20 ± 76.33	< 0.001
Fasting blood glucose, mg/dL	131.88 ± 53.33	126.83 ± 45.99	133.14 ± 51.00	0.006
Total cholesterol, mg/dL	168.18 ± 46.07	166.65 ± 38.01	168.51 ± 39.32	0.186
Triglyceride, mg/dL	155.53 ± 75.81	168.38 ± 82.93	168.58 ± 84.86	< 0.001
HDL cholesterol, mg/dL	49.41 ± 13.16	51.24 ± 13.47	49.78 ± 13.03	< 0.001
LDL cholesterol, mg/dL	97.60 ± 32.96	96.58 ± 31.68	97.53 ± 32.61	0.464
Creatinine, mg/dL	0.84 ± 0.66	0.83 ± 0.52	0.77 ± 0.35	< 0.001
AST, U/L	27.74 ± 15.40	26.87 ± 15.82	26.84 ± 15.64	0.066
ALT, U/L	28.67 ± 20.91	26.46 ± 19.80	27.64 ± 22.02	< 0.001
Body composition				
BMI, kg/m^2^	25.65 ± 3.87	25.59 ± 4.61	25.67 ± 3.76	0.770
SMM (kg)	25.85 ± 7.84	25.38 ± 5.78	24.60 ± 5.73	< 0.001
ASM (kg)	19.83 ± 5.06	19.69 ± 10.46	20.67 ± 5.22	0.295
SMI (kg/m^2^)	9.63 ± 2.43	9.39 ± 2.20	9.53 ± 1.31	< 0.001
RASM (kg/m^2^)	7.32 ± 1.22	7.41 ± 3.64	7.51 ± 1.21	0.540
Total fat %	30.82 ± 8.50	31.20 ± 8.19	31.74 ± 8.62	0.000
FMI (kg/m^2^)	8.10 ± 3.15	8.32 ± 3.25	8.20 ± 3.09	0.037
VFA (m^2^)	119.97 ± 29.46	118.98 ± 30.01	118.30 ± 35.64	0.322
VFI	44.24 ± 14.33	45.55 ± 12.33	46.05 ± 13.25	0.275
aMFR	1.05 ± 0.92	1.45 ± 5.47	1.08 ± 0.97	0.070
tMFR	2.64 ± 4.19	2.46 ± 2.16	3.24 ± 3.70	0.022
Co‐morbid conditions	2175 (80.89)	2428 (86.84)	2146 (78.35)	< 0.001
Hypertension	934 (34.73)	1125 (40.24)	1019 (37.20)	< 0.001
Dyslipidaemia	1241 (46.15)	1303 (46.60)	1127 (41.15)	< 0.001
Kidney function (eGFR)				
≥ 60 (Normal/Mild)	2585 (96.10)	2688 (96.10)	2595 (94.70)	< 0.001
30–59 (Moderate)	80 (2.97)	88 (3.15)	105 (3.83)	< 0.001
< 30 (Severe)	24 (0.89)	20 (0.72)	39 (1.42)	< 0.001
Macrovascular complications	9 (0.33)	16 (0.57)	7 (0.26)	0.210
Stroke	0 (0.00)	0 (0.00)	0 (0.00)	1.000
Dementia	3 (0.11)	6 (0.21)	1 (0.04)	0.162
Parkinson's disease	1 (0.04)	10 (0.36)	6 (0.22)	0.033
Peripheral vascular disease	2 (0.07)	0 (0.00)	0 (0.00)	0.128
Lower limb amputation	3 (0.11)	0 (0.00)	0 (0.00)	0.046
Microvascular complications	944 (35.11)	1119 (40.02)	1225 (44.72)	< 0.001
Retinopathy	188 (6.99)	220 (7.87)	297 (10.84)	< 0.001
Proliferative diabetic retinopathy	61 (2.27)	41 (1.47)	61 (2.23)	0.055
Chronic kidney disease	134 (4.98)	116 (4.15)	95 (3.47)	0.021
Neuropathy	561 (20.86)	742 (26.54)	772 (28.19)	< 0.001
Cancer	193 (7.18)	177 (6.33)	171 (6.24)	0.309
Medication use				
Diabetes mellitus	5501	5585	6349	0.015
Metformin	1895 (70.47)	1878 (67.17)	2097 (76.56)	< 0.001
Sulfonylurea	866 (45.70)	1012 (53.89)	1275 (60.80)	< 0.001
DPP‐4 inhibitor	1187 (44,14)	971 (34.73)	995 (36.33)	< 0.001
Meglitinide	84 (3.12)	125 (4.47)	169 (6.17)	< 0.001
Thiazolidinedione	169 (6.28)	249 (8.91)	282 (10.30)	< 0.001
*α*‐Glucosidase inhibitor	48 (1.79)	86 (3.08)	121 (4.42)	< 0.001
Insulin	1059 (39.38)	1126 (40.27)	1334 (48.70)	< 0.001
GLP‐1 receptor agonist	16 (0.60)	20 (0.72)	13 (0.47)	0.508
SGLT2 inhibitor	177 (6.58)	118 (4.22)	63 (2.30)	< 0.001
Hypertension	2633	3154	2950	0.048
Angiotensin II receptor blocker	982 (36.52)	1155 (41.31)	1165 (42.53)	< 0.001
ACE inhibitor	92 (3.42)	175 (6.26)	179 (6.54)	< 0.001
Calcium channel blocker	858 (31.91)	936 (33.48)	834 (30.45)	0.054
Diuretics	445 (16.55)	531 (18.99)	474 (17.31)	0.052
Beta‐blocker	256 (9.52)	357 (12.77)	298 (10.88)	0.001
Dyslipidaemia	1908	2135	2123	0.031
Statin	1549 (57.61)	1782 (63.73)	1754 (64.04)	< 0.001
Fibrate	124 (4.61)	137 (4.90)	153 (5.59)	0.240
Ezetimibe	156 (5.80)	102 (3.65)	74 (2.70)	< 0.001
Omega‐3	77 (2.86)	100 (3.58)	132 (4.82)	0.001
ETC dyslipidaemia	2 (0.07)	14 (0.50)	10 (0.37)	0.016
Antiplatelet	1377	2012	2111	< 0.001
Aspirin	405 (15.06)	621 (22.21)	609 (22.23)	< 0.001
Clopidogrel	303 (11.27)	401 (14.34)	406 (14.82)	< 0.001
Cilostazol	423 (15.73)	649 (23.21)	756 (27.60)	< 0.001
Glycoprotein IIb/IIIa antagonist	0 (0.00)	0 (0.00)	1 (0.04)	0.367
ETC antiplatelet^b^	246 (9.15)	341 (12.20)	339 (12.38)	< 0.001

*Note:* Values are presented as means ± standard deviation (SD) for continuous variables and the number (%) for categorical variables. Comparisons of continuous variables were performed by one‐way analysis of variance, and Kruskal–Wallis was used to compare categorical variables.

^a^ HbA1c variability score (HVS), calculated by the number of successive measurements that differed by 0.5% (5.5 mmol/mol) or more, divided by the number of comparisons and then multiplied by 100.

^b^ ETC stands for ‘et cetera’ and is used here to mean ‘other types of’.

Abbreviations: ALP, alkaline phosphatase; ALT, Alanine transaminase; aMFR, appendicular muscle mass/total body fat; ASM, appendicular skeletal muscle mass; AST, aspartate transaminase; BMI, body mass index; BP, blood pressure; DPP‐4, dipeptidyl peptidase‐4; HbA1c, glycated haemoglobin; eGFR, estimated glomerular filtration rate; FMI, total fat mass/height (m)^2^; FPG, fasting plasma glucose; GGT, gamma‐glutamyl transferase; GLP‐1, glucagon‐like peptide‐1; HDL, high‐density lipoprotein, LDL, low‐density lipoprotein; PP2hrs; postprandial 2‐h glucose; RASM, skeletal muscle mass (kg)/height (m)^2^; SGLT2, sodium‐glucose cotransporter 2; SMI, SMM/height (m)^2^; SMM, skeletal muscle mass; tMFR, total body mass (kg)/total body fat (kg); VFA, visceral fat area (m)^2^; VFI, visceral fat area/height (m)^2^.

### 3.2 Correlation Between Glucose, Body Composition, Their Changes and HVS

Baseline FPG and PP2Hr glucose levels positively correlated with HVS, with HbA1c demonstrating a stronger association than FPG and PP2Hr glucose (FPG, *β* = 0.061, *p* < 0.001; PP2Hr, *β* = 0.088, *p* < 0.001; HbA1c, *β* = 0.237, *p* < 0.001). This correlation pattern was consistent for changes in the glucose parameters (ΔFPG, *β* = 0.272, *p* < 0.001; ΔPP2Hr, *β* = 0.309, *p* < 0.001; ΔHbA1c, *β* = 0.527, *p* < 0.001) (Table 2). Regarding body composition, baseline muscle‐related parameters exhibited a positive correlation with HVS, whereas fat‐related parameters demonstrated a negative correlation; however, these associations were not statistically significant. Notably, the tMFR revealed a significant positive correlation with HVS (*β* = 0.054, *p* = 0.002). Alterations in muscle‐related indicators such as SMM, ASM, SMI and RASM were negatively correlated with the HVS. In contrast, changes in fat‐related indicators such as FMI, VFA and total body fat percentage were positively correlated with the HVS, tMFR and aMFR. Among body composition components, RASM exhibited a significant negative correlation, while tMFR demonstrated a significant positive correlation with HVS for both men and women (ΔRASM, *β* = −0.268, *p* < 0.001 in men; *β* = −0.248, *p* < 0.001 in women; ΔtMFR, *β* = 0.326, *p* < 0.001 in men; *β* = 0.381, *p* < 0.001 in women) (Table 2).

**TABLE 2:** Sex differences in the Pearson correlation between HVS and changes in various parameters.

	Total (*n* = 8224)		Male (*n* = 4105)		Female (*n* = 4119)	
	Correlation coefficient	*p*	Correlation coefficient	*p*	Correlation coefficient	*p*
Age	−0.077	< 0.001	0.103	< 0.001	0.048	0.002
HbA1c	0.237	< 0.001	0.223	< 0.001	0.254	< 0.001
PP2hrs	0.088	< 0.001	0.101	< 0.001	0.073	0.000
FPG	0.061	< 0.001	0.075	0.002	0.043	0.075
BMI	−0.009	0.435	−0.014	0.382	−0.004	0.795
SMM	0.005	0.675	0.000	0.991	0.000	0.998
ASM	0.021	0.400	0.002	0.965	0.037	0.294
SMI	0.001	0.942	−0.005	0.731	0.012	0.432
RASM	0.010	0.697	−0.009	0.806	0.022	0.538
Total fat %	−0.017	0.130	−0.022	0.164	−0.009	0.549
FMI	−0.014	0.190	−0.021	0.174	−0.006	0.718
VFA	−0.023	0.361	−0.028	0.418	−0.017	0.627
VFI	−0.036	0.145	−0.037	0.286	−0.030	0.385
aMFR	−0.013	0.598	−0.011	0.742	−0.018	0.605
tMFR	0.054	0.002	0.030	0.265	−0.074	0.202
Δ Age	0.459	< 0.001	0.465	< 0.001	0.457	< 0.001
Δ HbA1c	0.527	< 0.001	0.543	< 0.001	0.511	< 0.001
Δ PP2hrs	0.309	< 0.001	0.302	< 0.001	0.316	< 0.001
Δ FPG	0.272	< 0.001	0.288	< 0.001	0.254	< 0.001
Δ BMI	0.464	< 0.001	0.484	< 0.001	0.445	< 0.001
Δ SMM	−0.458	< 0.001	−0.484	< 0.001	−0.448	< 0.001
Δ ASM	−0.254	< 0.001	−0.267	< 0.001	−0.247	< 0.001
Δ SMI	−0.472	< 0.001	−0.489	< 0.001	−0.455	< 0.001
Δ RASM	−0.258	< 0.001	−0.268	< 0.001	−0.248	< 0.001
Δ Total fat %	0.439	< 0.001	0.469	< 0.001	0.447	< 0.001
Δ FMI	0.399	< 0.001	0.430	< 0.001	0.403	< 0.001
Δ VFA	0.249	< 0.001	0.256	< 0.001	0.243	< 0.001
Δ VFI	0.244	< 0.001	0.254	< 0.001	0.241	< 0.001
Δ aMFR	0.219	< 0.001	0.225	< 0.001	0.242	< 0.001
Δ tMFR	0.342	< 0.001	0.326	< 0.001	0.381	< 0.001

*Note:* HbA1c variability score (HVS), calculated by the number of successive measurements which were differed by 0.5% (5.5 mmol/mol) or more, divided by the number of comparisons and then multiplied by 100.

Abbreviations: aMFR, appendicular muscle mass/total body fat; ASM, appendicular skeletal muscle mass; BMI, body mass index; FMI; total fat mass/height (m)^2^; FPG, fasting plasma glucose; HbA1c, glycated haemoglobin; PP2hrs, postprandial 2‐h glucose; RASM, skeletal muscle mass (kg)/height (m)^2^; SMI, SMM/height (m)^2^; SMM, skeletal muscle mass; tMFR, total body mass (kg)/total body fat (kg); VFA, visceral fat area (m)2; VFI, visceral fat area/height (m)^2^.

### 3.3 Assessment of Risk Factors for CVD

Univariate Cox regression analysis was used to identify multiple risk factors for CVD. Individuals aged ≥ 65 years (hazard ratio [HR]: 1.62, 95% confidence interval [CI]: 1.11–2.36), those with variability in HbA1c levels (HR: 1.21, 95% CI: 1.18–1.30) and individuals with hyperlipidemia (HR: 1.20, 95% CI: 1.00–1.44) were at increased risk of CVD. Among body composition parameters, TFP (HR: 1.01, 95% CI: 1.00–1.02) and FMI (HR 1.03, 95% CI 1.00–1.06) were associated with elevated CVD risk, while increased ASM was associated with a reduced risk of CVD (HR: 0.75, 95% CI: 0.64–0.87). Furthermore, changes in TFP (HR: 1.30, 95% CI: 1.15–1.47) and tMFR (HR: 1.11, 95% CI: 1.03–1.39) were associated with increased risk of CVD (Table 3).

**TABLE 3:** Univariate Cox regression for the HRs of CVD risk.

Covariates	HRs	95% CI	*p*
Age, years			
20–39	Ref.		
40–64	1.03	0.73, 1.47	0.857
≥ 65	1.62	1.11, 2.36	0.012
Sex			
Male	Ref.		
Female	0.98	0.82, 1.16	0.785
Glucose			
HbA1c	1.062	0.99, 1.14	0.094
Δ HbA1c	1.21	1.18, 1.30	< 0.001
Hypertension			
No	Ref.		
Yes	1.20	1.10, 1.43	0.051
Dyslipidaemia			
No	Ref.		
Yes	1.20	1.00, 1.44	0.046
Glucose‐lowering medication			
No	Ref.		
Yes	1.26	0.96, 1.66	0.092
Body composition	Ref.		
SMM	1.01	0.98, 1.03	0.614
ASM	0.93	0.86, 1.01	0.080
SMI	1.15	0.59, 2.24	0.689
RASM	0.87	0.45, 1.70	0.684
Total fat %	1.01	1.00, 1.02	0.040
FMI	1.03	1.00, 1.06	0.032
VFA	1.02	0.89, 1.30	0.693
VFI	1.00	0.98, 1.02	0.760
aMFR	1.05	0.82, 1.33	0.720
tMFR	1.00	0.95, 1.06	0.876
Δ Body composition			
Δ SMM	1.03	1.00, 1.05	0.038
Δ ASM	0.75	0.64, 0.87	< 0.001
Δ SMI	1.76	1.00, 2.01	0.049
Δ RASM	0.94	0.87, 1.11	0.107
Δ Total fat %	1.30	1.15, 1.47	0.002
Δ FMI	1.03	1.00, 1.06	0.032
Δ VFA	0.99	0.79, 1.37	0.240
Δ VFI	1.01	0.84, 1.26	0.558
Δ aMFR	1.08	0.81, 1.65	0.073
Δ tMFR	1.11	1.03, 1.39	< 0.001

*Note:* Cardiovascular diseases (CVD) include ischemic heart disease, heart failure, atrial fibrillation, myocardial infarction and stroke.

Abbreviations: aMFR, appendicular muscle mass/total body fat; ASM, appendicular skeletal muscle mass; CI, confidence interval; FMI, total fat mass/height (m)^2^; HbA1c; glycated haemoglobin, HR, hazard ratio; RASM, skeletal muscle mass (kg)/height (m)^2^; SMI, SMM/height (m)^2^; SMM, skeletal muscle mass; tMFR, total body mass (kg)/total body fat (kg); VFA, visceral fat area (m)^2^; VFI, visceral fat area/height (m)^2^.

### 3.4 Relationship Between Glucose Variability, Body Composition, and the Risk of CVD

**FIGURE 2:**
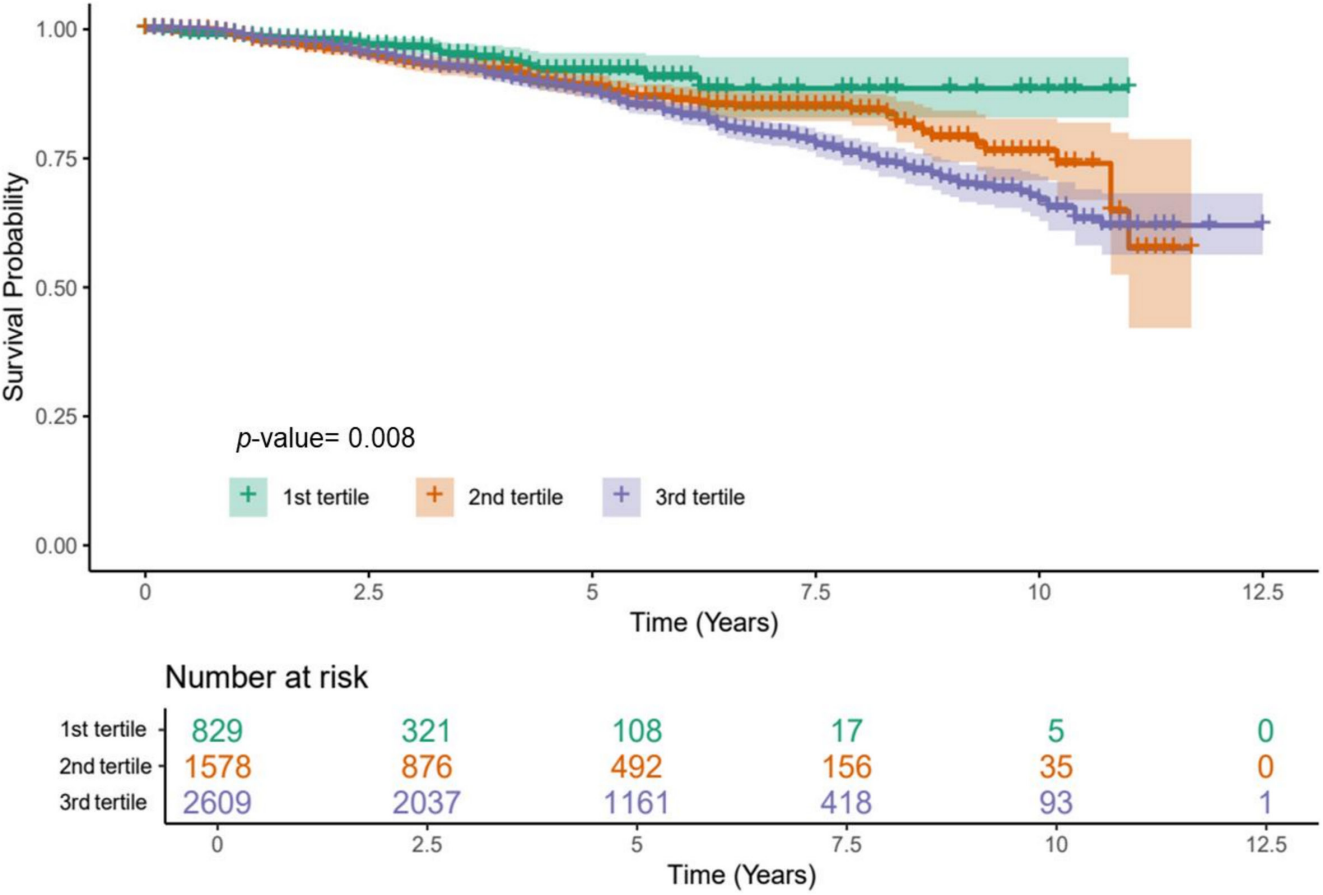
The Kaplan–Meier curve for glycated haemoglobin variability score and overall survival. Glycated haemoglobin variability score was calculated as the number of successive measurements that differed by 0.5% (5.5 mmol/mol) or more, divided by the number of comparisons, and then multiplied by 100.

**FIGURE 3:**
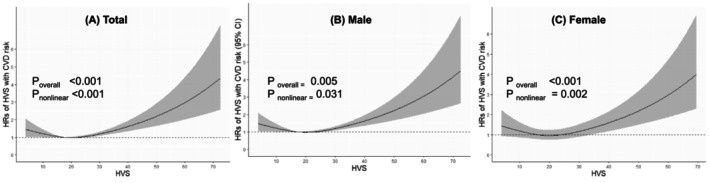
Cubic spline analysis of hazard ratios with glycated haemoglobin (HbA1c) variability score for cardiovascular disease risk in total (A), men (B) and women (C) adjusted for interval mean HbA1c. HbA1c variability score (HVS) calculated by the number of successive measurements, which differed by 0.5% (5.5 mmol/mol) or more, divided by the number of comparisons and then multiplied by 100.

Higher glucose variability is associated with an increased CVD risk. Notably, HbA1c variability demonstrated a more significant correlation with CVD risk among the glucose variability metrics than FPG and PP2hr variability (Figure 2). The risk of CVD gradually increased within the HVS range of 20–40 and rose sharply when the HVS exceeded 40, a trend consistent across both sexes (Figure 3).

While FPG and PP2hr variability did not significantly correlate with increased CVD risk, HbA1c variability was significantly associated with an elevated risk of CVD in the third tertile after adjusting for age, sex, HbA1c, hypertension, dyslipidaemia, GLM and eGFR (Model 2) (adjusted HR [aHR]: 1.52, 95% CI: 1.01–2.08; P for trend 0.070). The risk of CVD significantly increased with each tertile increase in HVS, even after adjusting for additional body composition factors (Model 3, further adjusted for ASM, aHR: 1.60, 95% CI: 1.05–2.44; Model 4, further adjusted for TFP, aHR: 1.70, 95% CI: 1.13–2.40) (Table 4).

**TABLE 4:** HRs and 95% CIs for risk of CVD according to the tertiles of HVS score groups.

	1st tertile	2nd tertile	3rd tertile	*p*	*p* trend
FPG variability groups					
Cases/non‐cases	28/584	23/580	36/558		
Model 1	1 (Reference)	0.91 (0.52, 1.58)	1.39 (0.82, 2.34)	0.257	0.565
Model 2	1 (Reference)	1.03 (0.76, 1.40)	1.45 (0.95, 2.02)	0.090	0.102
Model 3	1 (Reference)	1.01 (0.74, 1.38)	1.47 (0.98, 2.01)	0.130	0.088
Model 4	1 (Reference)	0.98 (0.72, 1.30)	1.52 (1.01, 2.08)	0.070	0.059
PP2hrs (PPG) variability groups					
Cases/non‐cases	78/1247	82/1284	80/1241		
Model 1	1 (Reference)	0.97 (0.70, 1.33)	1.03 (0.75, 1.42)	0.097	0.071
Model 2	1 (Reference)	1.12 (0.70, 1.78)	1.55 (1.00, 2.45)	0.095	0.081
Model 3	1 (Reference)	1.13 (0.75, 1.74)	1.57 (1.03, 2.39)	0.106	0.074
Model 4	1 (Reference)	1.15 (0.77, 1.71)	1.60 (1.05, 2.44)	0.085	0.069
HVS					
Cases/non‐cases	68/2621	143/2653	389/2350		
Model 1	1 (Reference)	1.40 (0.94, 2.09)	1.69 (1.15, 2.47)	0.009	0.005
Model 2	1 (Reference)	1.42 (0.94, 2.18)	1.64 (1.12, 2.40)	0.007	0.010
Model 3	1 (Reference)	1.40 (0.93, 2.09)	1.63 (1.11, 2.39)	0.014	< 0.001
Model 4	1 (Reference)	1.40 (0.95, 2.10)	1.70 (1.13, 2.40)	< 0.001	< 0.001

*Note:* Cardiovascular diseases (CVD) include ischemic heart disease, heart failure, atrial fibrillation, myocardial infarction and stroke. HbA1c variability score (HVS) is calculated by the number of successive measurements that differed by 0.5% (5.5 mmol/mol) or more divided by the number of comparisons and then multiplied by 100. The *p* trend was assessed by treating the categorical variable as continuous.

Abbreviations: CI, confidence interval; FPG, fasting plasma glucose; HbA1c, glycated haemoglobin; HR, hazard ratio; PP2hrs, postprandial 2‐h glucose.

Model 1 non‐adjusted;

Model 2 age, sex, HbA1c, HTN, dyslipidaemia, glucose‐lowering medication, estimated glomerular filtration rate;

Model 3 model 2 + Δ aASM;

Model 4 model 2 + Δ Total fat %.

**FIGURE 4:**
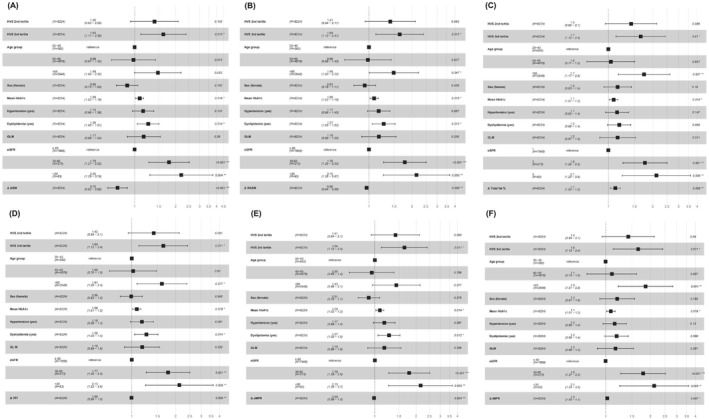
HRs (95% CI) for CVD risk stratified by clinical and demographic characteristics. GLMs, glucose‐lowering medications; eGFR, estimated glomerular filtration rate; SMM, skeletal muscle mass; ASM, skeletal muscle mass; SMI, SMM/height^2^; RASM, appendicular skeletal muscle mass divided by squared body height^2^; FMI, fat mass index; VFA, visceral fat area; VFI, VFA/height^2^; aMFR, ASM/total body fat mass; tMFR, total body muscle mass/total body fat mass. (A) adjusted age, sex, HbA1c, history of hypertension, dyslipidaemia, eGFR, GLMs + Δ ASM; (B) adjusted age, sex, HbA1c, history of hypertension, dyslipidaemia, eGFR, GLMs + Δ RASM; (C) adjusted age, sex, HbA1c, history of hypertension, dyslipidaemia, eGFR, GLMs + Δ Total fat %; (D) adjusted age, sex, HbA1c, history of hypertension, dyslipidaemia, eGFR, GLMs + Δ VFI; (E) adjusted age, sex, HbA1c, history of hypertension, dyslipidaemia, eGFR, GLMs + Δ aMFR; (F) adjusted age, sex, HbA1c, history of hypertension, dyslipidaemia, eGFR, GLMs + Δ tMFR.

Analysis of the impact of various body composition components on CVD risk showed that the risk was lower when muscle‐related variables were adjusted for baseline factors. (Figure 4 and Supporting Information S1). Notably, the adjustment for ASM significantly reduces CVD risk, resulting in aHR of 0.75 (95% CI: 0.63–0.88) as shown in Figure 4A. When additional adjustments are made for fat‐related factors, particularly the TFP, the hazard ratio for CVD increases to 1.10 (95% CI: 1.03–1.20), indicating a higher risk (Figure 4C).

When all changes in body composition are simultaneously adjusted, significant contributors to increased CVD risk include the third quartile of HVS (aHR 1.69, 95% CI: 1.15–2.50, *p* = 0.007), elevated average HbA1c levels (aHR 1.08, 95% CI: 1.01–1.20, *p* = 0.026) and individuals aged over 65 (HR 1.58; *p* = 0.049). (Supporting Information S2).

## 4 Discussion

### 4.1 Key Findings

This study provides critical insights into the interplay between glycaemic variability, body composition and cardiovascular outcomes in patients with T2DM. Using a large cohort of 8224 individuals with T2DM, we demonstrated that high HbA1c variability is a significant predictor of CVD, including cardiovascular mortality. Specifically, individuals in the highest HVS tertile had a 70% higher risk of developing CVD compared to those in the lowest tertile. This finding underscores the prognostic importance of glycaemic stability beyond absolute glycaemic levels. Besides, higher ASM protects against CVD, reducing risk by 25% among people with T2DM, while increases in TFP are linked to a 10% heightened risk of CVD. Moreover, a higher muscle‐to‐fat ratio (tMFR), a key indicator of body composition, is associated with improved cardiovascular outcomes, further emphasizing the critical role of maintaining a favourable muscle‐to‐fat balance in the comprehensive assessment of cardiovascular risk. Incorporating body composition metrics and glycaemic variability into established indicators of diabetes care quality may enhance the prediction and management of CVD risk in individuals with T2DM.

### 4.2 Comparisons With Previous Studies

The impact of glycaemic control on microvascular complications, including retinopathy, nephropathy, and neuropathy, in persons with T2DM has been extensively reported in the literature [2, 21]. However, the long‐term impact of glycaemic control on the full spectrum of macrovascular disease remains uncertain, with existing long‐term follow‐up studies primarily demonstrating reductions in myocardial infarction and all‐cause mortality [22]. The uncertainty stems from the complex interplay of various risk factors contributing to CVD events in persons with T2DM [23]. Recent studies have demonstrated that the HbA1c is a superior predictor of CVD risk compared to the glycaemic index, highlighting its significance as an important prognostic marker in cardiovascular risk management [24, 25]. In the present study, after adjusting for various confounding factors, we found that higher (HVS) was significantly associated with an increased risk of CVD, suggesting that glycaemic variability also plays a critical role in cardiovascular outcomes. Therefore, comprehensive monitoring of both the HbA1c level and its variability is warranted to optimize cardiovascular risk assessment and management strategies in patients with T2DM.

Glycaemic variability has gained extensive attention in association with diabetes care, whether it is continuous glucose monitoring (CGM) or visit‐to‐visit HbA1c [26]. A recent review summarizes evidence from 34 studies demonstrating an association between CGM‐derived measures of glycaemic variability, particularly low time in range, and a higher risk of microvascular and macrovascular complications in people with DM [27]. Meta‐analysis of 146 653 individuals with T2DM demonstrated that increased long‐term glycemic variability, as measured by standard deviation and CV of HbA1c and fasting plasma glucose, was significantly associated with an increased risk of cardiovascular events [28]. This study demonstrates a robust association between increased HVS and an elevated risk of CVD in individuals with T2DM. These findings underscore the critical importance of achieving glycemic stability in managing T2DM and suggest that the HVS should be considered a valuable parameter in guiding individualized treatment strategies for this population.

Few studies have investigated the association between body composition and incident CVD in individuals with T2DM. A study analysing the UK Biobank reported that body fat exacerbates CVD risk, a finding also corroborated by various anthropometric measures of adiposity [29, 30]. Other studies have reported that moderate‐to‐strong correlations between body fat percentage and CVD risk factors are consistent with prior research, which demonstrated that fat mass significantly increases the risk of CVD, with notable findings indicating that temporal changes in fat mass are more strongly associated with an elevated risk of CVD [4]. Although limited research is available on ASM, some studies have demonstrated an inverse correlation between ASM and CVD risk. In the ATTICA study of 1019 CVD‐free adults aged 45 years and older, lower skeletal muscle mass was significantly associated with a higher 10‐year risk of CVD, independent of traditional risk factors, suggesting that maintaining skeletal muscle mass may be crucial for cardiovascular health [31]. This study observed an inverse correlation between muscle mass and HVS variability, while changes in ASM were linked to a reduced risk of CVD.

A prior study investigated the relationship between skeletal muscle mass and glycemic variability in 208 patients with myocardial infarction using CGM and found a significant inverse association between skeletal muscle mass and glycemic variability, independent of traditional risk factors like HbA1c and BMI, suggesting that increased skeletal muscle mass may contribute to improved glucose control by enhancing glucose storage and potentially reducing glycaemic fluctuations [32]. Given that low skeletal muscle mass and elevated fat mass constitute established risk factors for VD, a composite muscle‐to‐fat ratio has been developed and employed as a surrogate marker for whole‐body muscle‐to‐fat balance, demonstrating prognostic utility for cardiometabolic outcomes [33]. As a potential biomarker of sarcopenic obesity, both aMFR and tMFR effectively differentiate cardiovascular risk profiles in older adults, with tMFR demonstrating superior predictive capability [18]. This study further supports these findings, demonstrating a positive correlation between tMFR and HVS. Additionally, changes in tMFR were positively correlated with the HVS across the overall and sex‐specific analyses, underscoring its significance in increasing CVD risk. However, multivariate analysis revealed that aMFR, rather than tMFR, was associated with a preventive effect by reducing the risk of CVD. The superiority of aMFR over tMFR suggests the greater importance of the appendicular muscle mass, which is more closely associated with sarcopenia and exercise‐related effects, warranting further investigation.

### 4.3 Possible Explanations

Individuals with T2DM typically exhibit a phenotype characterized by abdominal obesity and reduced thigh circumference [34]. Visceral adiposity plays an integral role in the pathophysiological mechanisms underlying diabetes and its associated complications [35], while skeletal muscle is crucial for glucose uptake and systemic metabolic regulation [36]. Furthermore, elevated blood glucose levels, particularly with increased glycemic variability, can adversely affect various molecular mechanisms in multiple target cells and tissues, primarily contributing to microvascular and macrovascular complications in individuals with diabetes [37]. At the macrovascular level, hyperglycemia induces inflammation, thrombosis, protein glycation, and reactive oxygen species formation, which are key in atherosclerosis development. Small blood vessels respond to hyperglycemia‐induced hypoxia and ischemia through angiogenesis, linking microvascular and macrovascular pathologies [38].

### 4.4 Limitations and Strengths

Despite extensive research, this study has several limitations. First, the Kyung Hee University EHR data did not include information on lifestyle factors, such as alcohol consumption, smoking and physical activity. The American Diabetes Association emphasizes that alongside glycaemic management, physical activity and dietary interventions significantly influence the occurrence of diabetes complications and the risk of CVD [39]. However, we exclusively analysed individuals with T2DM treated with GLM, excluding those managed solely with lifestyle modifications, to ensure robust validation of EHR‐based T2DM diagnoses. Focusing on individuals with T2DM treated with the GLM ensures homogeneity among study participants and enhances the applicability of the findings within clinical settings. Second, we used BIA rather than computed tomography or magnetic resonance imaging for body composition assessment, which are considered gold standards. In contrast, BIA offers a convenient and noninvasive alternative for relatively accurate estimation without radiation exposure, typically within a five‐minute timeframe. BIA is a validated non‐invasive tool for assessing body composition in clinical practice and large‐scale population‐based studies, as evidenced by its effectiveness compared to DXA in a recent study of UK Biobank participants [40]. Third, this study utilized data from a single institution, which may limit the generalizability of the findings to broader populations. Nevertheless, using data from a single institution allowed for consistent data collection and standardized clinical protocols, ensuring high‐quality, reliable data for analysis. Future research involving multicenter or population‐based datasets must validate these findings in diverse settings. Fourth, due to limitations in the EHR database, we were unable to include certain potentially important confounders, such as the duration of diabetes and socioeconomic status (e.g., income, education or insurance type), which have been shown to influence cardiovascular outcomes in patients with T2DM. To partially address this limitation, we incorporated renal function into our models by including estimated glomerular filtration rate (eGFR), a well‐established clinical marker of diabetes progression and a strong independent predictor of cardiovascular risk. Prior studies have consistently demonstrated that reduced eGFR is associated with increased cardiovascular morbidity and mortality in individuals with diabetes [41, 42]. Therefore, while direct information on diabetes duration and socioeconomic status was not available, the inclusion of eGFR allowed us to account for underlying disease burden to some extent.

Despite these limitations, this study has several strengths. First*,* the diagnosis of T2DM in this cohort was based on previously validated EHR studies [43]. Second, this research provided robust data selection and processing with a follow‐up of up to 11 years, ensuring high‐quality longitudinal data. Third, the findings regarding the significance of HbA1c variability and aMFR in predicting CVD risk in adults with T2DM beyond traditional risk factors provide novel evidence for improving diabetes care, congruent with the objectives of healthy ageing.

### 4.5 Future Directions

Future research should prioritize refining predictive models that incorporate HbA1c variability and body composition indicators to enhance the accuracy of cardiovascular risk assessments. These models serve as critical tools for evaluating individual health statuses with precision, thereby facilitating the development of personalized treatment plans. By exploring additional biomarkers to complement these indicators, researchers can further improve the precision of risk predictions. Additionally, rigorous studies are necessary to evaluate the efficacy of interventions aimed at reducing CVD risk in vulnerable populations. Specifically, assessing the effectiveness of interventions that reduce HbA1c variability and improve body composition will determine their applicability in clinical settings. Such evaluations are crucial to ensure these interventions can be effectively implemented in real‐world healthcare environments.

### 4.6 Clinical Implications

This study underscores the pivotal role of HbA1c variability and body composition indicators in forecasting CVD risk among patients with T2DM. By evaluating these parameters, healthcare providers can formulate personalized treatment plans that significantly enhance patient outcomes. The integration of HbA1c variability and body composition metrics facilitates a more precise stratification of CVD risk, promoting the development of tailored strategies that encompass both pharmacological interventions and lifestyle modifications. This approach aligns with the objectives of personalized medicine, aiming to optimize therapeutic outcomes and increase patient satisfaction.

Moreover, the findings of this study offer new evidence that could inform updates to clinical guidelines for managing T2DM and associated cardiovascular risks, potentially leading to more effective strategies for preventing cardiovascular events within this population. The study also aligns with the goals of healthy ageing, indicating that integrating these indicators into diabetes management can reduce the burden of CVD as patients age, thereby contributing to improved ageing outcomes.

## 5 Conclusion

The study identified that HbA1c variability and body composition independently predicted cardiovascular outcomes in patients with T2DM. Higher HbA1c variability increased CVD risk by 70%, while higher appendicular muscle mass reduced risk by 25%, and higher TFP increased risk by 10%. These findings suggest that integrating body composition indicators and glycemic variability into standard diabetes management could enhance the prediction and management of cardiovascular risk in patients with T2DM.

## Ethics Statement

This study was approved by the Institutional Review Board of Kyung Hee University Hospital (No. KHUH 2023‐11‐075). The Institutional Review Board waived the requirement for informed consent because de‐identified data were used for the analyses, and the research was performed following the relevant guidelines, regulations and the Declaration of Helsinki.

## Conflicts of Interest

The authors declare no conflicts of interest.

## Supporting information


**Figure S1.** HRs (95% CI) for CVD risk stratified by clinical and demographic characteristics.
**Figure S2.** HRs (95% CI) for CVD risk stratified by clinical and demographic characteristics.
