# Supplementary material for: Dual Roles of HbA1c Variability and Body Composition for Cardiovascular Risk: A Cohort Study of 8224 Adults With Type 2 Diabetes Mellitus
Source: J Cachexia Sarcopenia Muscle. 2025 Jul 29;16(4):e70028. doi: 10.1002/jcsm.70028 (PMC12304735; doi:10.1002/jcsm.70028)
Supplement: Supplementary file 1 — Figure S1. HRs (95% CI) for CVD risk stratified by clinical and demographic characteristics. Figure S2. HRs (95% CI) for CVD risk stratified by clinical and demographic characteristics. [file JCSM-16-e70028-s001.docx]

**Supplementary Figure 1.** HRs (95% CI) for CVD risk stratified by clinical and demographic characteristics


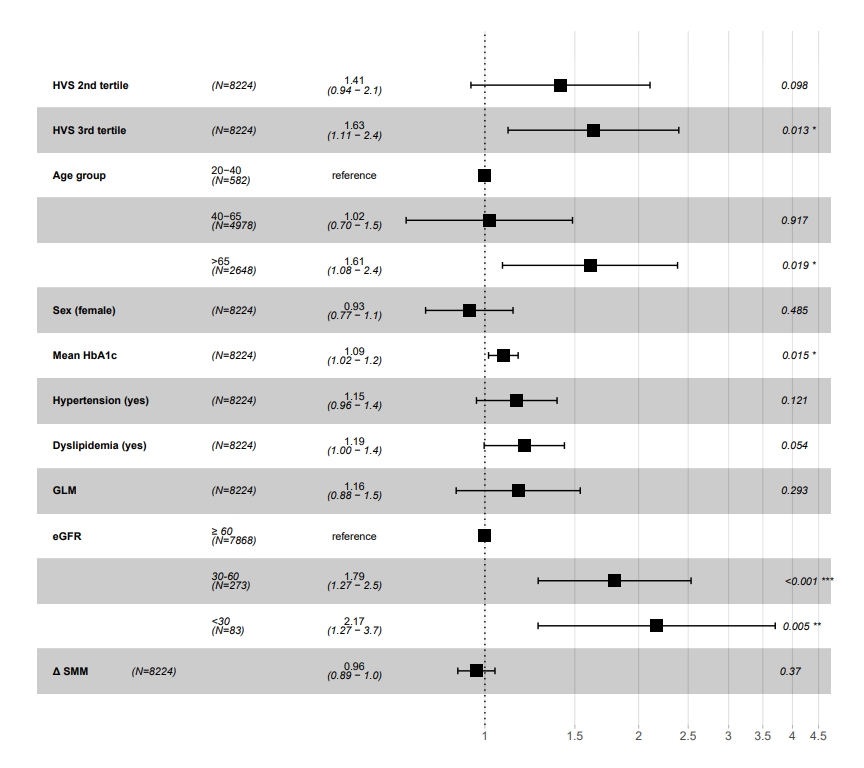


**(A)**


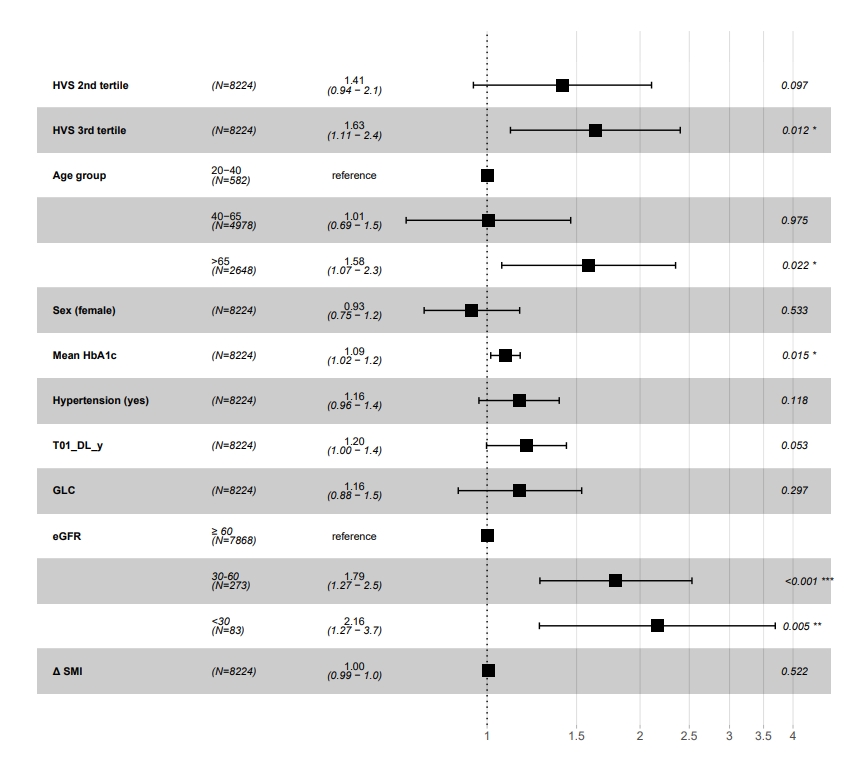


**(B)**


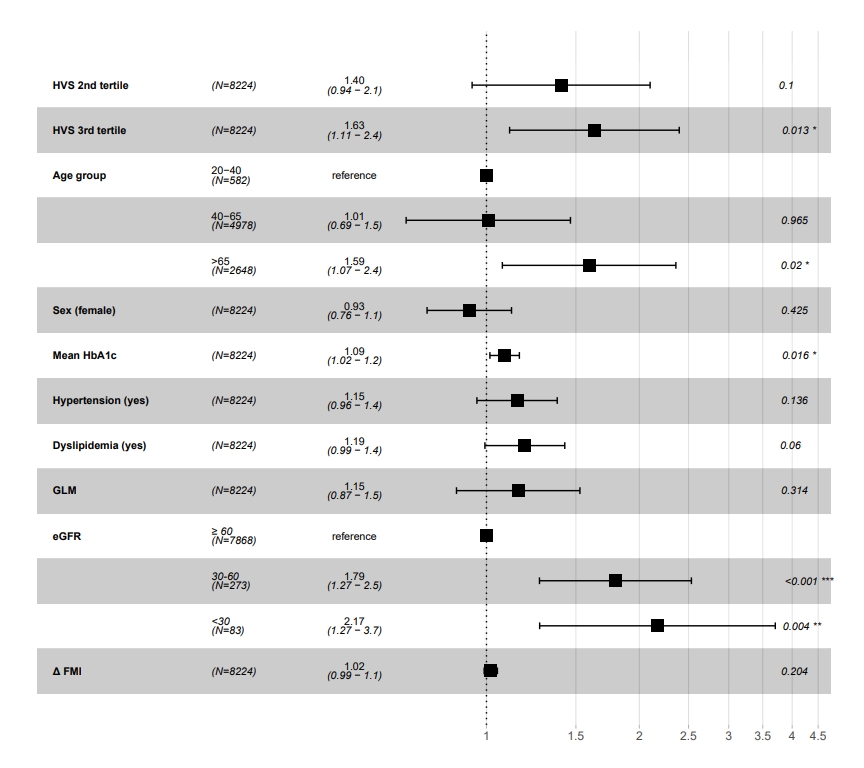


**(D)**

**(C)**


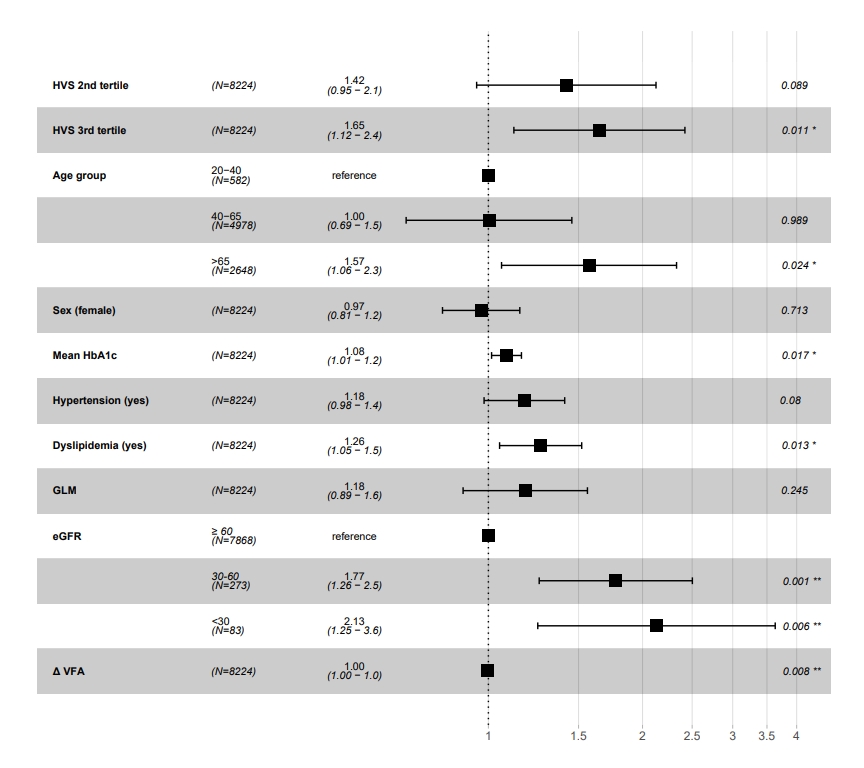


GLMs, glucose-lowering medications; eGFR, estimated glomerular filtration rate; SMM, skeletal muscle mass; ASM, skeletal muscle mass; SMI, SMM/height²; RASM, appendicular skeletal muscle mass divided by squared body height^2^; FMI, fat mass index; VFA, visceral fat area; VFI, VFA/height²; aMFR, ASM/total body fat mass; tMFR, total body muscle mass/total body fat mass.

(A) adjusted age, sex, HbA1c, history of hypertension, dyslipidemia, eGFR, GLMs + Δ SMM;

(B) adjusted age, sex, HbA1c, history of hypertension, dyslipidemia, eGFR, GLMs + Δ SMI;

(C) adjusted age, sex, HbA1c, history of hypertension, dyslipidemia, eGFR, GLMs + Δ FMI;

(D) adjusted age, sex, HbA1c, history of hypertension, dyslipidemia, eGFR, GLMs + Δ VFA;

**Supplementary Figure 2** HRs (95% CI) for CVD risk stratified by clinical and demographic characteristics


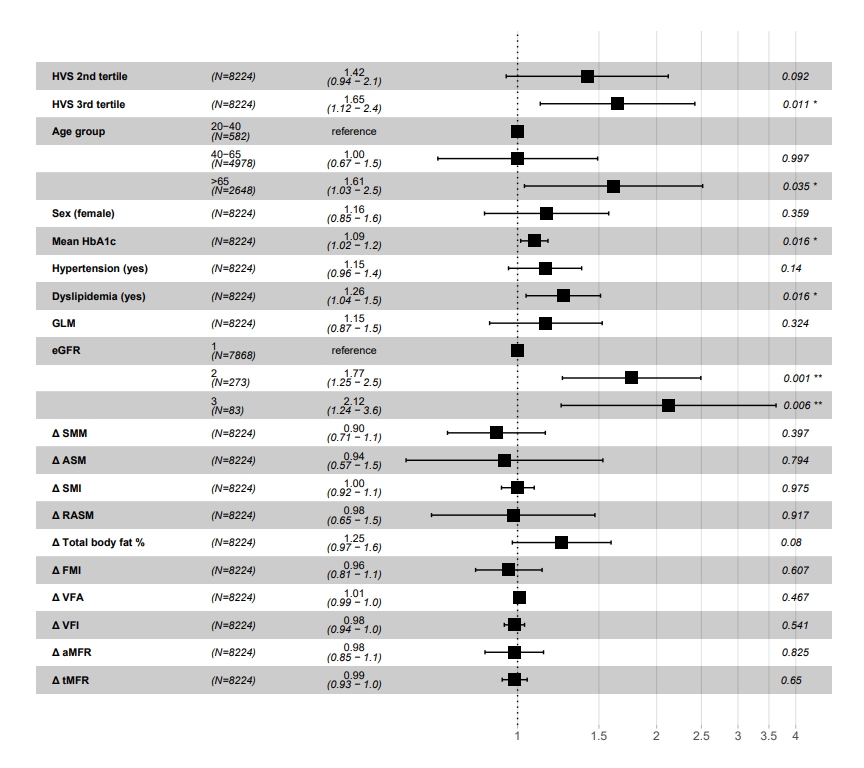


This model was adjusted age, sex, HbA1c, history of hypertension, dyslipidemia, GLMs, eGFR + Δ SMM + Δ ASM + Δ SMI + Δ RASM + Δ Total fat % + Δ FMI + Δ VFA + Δ VFI + Δ aMFR + Δ tMFR. eGFR, estimated glomerular filtration rate; GLMs, glucose-lowering medications; SMM, skeletal muscle mass; ASM, skeletal muscle mass; SMI, SMM/height²; RASM, appendicular skeletal muscle mass divided by squared body height^2^; FMI, fat mass index; VFA, visceral fat area; VFI, VFA/height²; aMFR, ASM/total body fat mass; tMFR, total body muscle mass/total body fat mass.
